# Overview of Drug Transporters in Human Placenta

**DOI:** 10.3390/ijms222313149

**Published:** 2021-12-05

**Authors:** Michiko Yamashita, Udo R. Markert

**Affiliations:** 1 Department of Obstetrics and Gynecology, Graduate School of Medicine, Osaka University, Osaka 5650871, Japan; 2 Placenta Lab, Department of Obstetrics, Jena University Hospital, Am Klinikum 1, 07747 Jena, Germany; udo.markert@med.uni-jena.de

**Keywords:** drug transporters, placenta, pregnancy, placental transport, membrane transporters, trophoblasts

## Abstract

The transport of drugs across the placenta is a point of great importance in pharmacotherapy during pregnancy. However, the knowledge of drug transport in pregnancy is mostly based on experimental clinical data, and the underlying biological mechanisms are not fully understood. In this review, we summarize the current knowledge of drug transporters in the human placenta. We only refer to human data since the placenta demonstrates great diversity among species. In addition, we describe the experimental models that have been used in human placental transport studies and discuss their availability. A better understanding of placental drug transporters will be beneficial for the health of pregnant women who need drug treatment and their fetuses.

## 1. Introduction

Transporters are membrane proteins that regulate the absorption, distribution, and excretion of substrates in all cells of the body. Transporters contribute to the creation of density gradients inside of and outside of membranes by actively importing and exporting a broad variety of substances. Although transporters were originally classified based on their functions, they are now classified according to the sequence homology of the isolated genes. Transporters are categorized into two superfamilies: ATP-binding cassette (ABC) transporters, which are involved in the efflux of substances, and solute carrier (SLC) transporters, which are mainly involved in the uptake of substances [1,2]. Transporters are involved in the transport of various physiological substances such as sugars, amino acids, and lipids, but some transporters are particularly relevant to pharmacokinetics and are thus recognized as drug transporters.

Drug transporters have two main roles: one is to control the uptake of drugs during their circulation from the organs that associated with alimentation, such as the small intestine, and their transport in excretory organs, such as the liver and kidneys. Their other role is to control local drug concentrations in order to protect critical cells, tissues, and organs from xenobiotics, such as at the blood–brain barrier, blood–cerebrospinal fluid barrier, and blood–placental barrier. The body is protected biologically from xenobiotics through the coexistence of ABC transporters and SLC transporters in each organ.

The placenta connects the fetus to the mother through the umbilical cord and supplies the fetus with nutrients and oxygen. Due to a history of harmful side effects on the fetus, the placental transport of drugs has always been an important issue. Regarding detoxification from xenobiotics, metabolic enzymes are involved [3], but transporters may have similar importance. This may be due to immature detoxification functions in the developing fetus or to an insufficiency of xenobiotic detoxifying capacities in the placental metabolic enzymes.

Thus, in this review, we summarize published data on human placental drug transporters and discuss the availability of experimental models. Animal data are not mentioned in this review because the placenta is an organ with great diversity among species not only regarding its construction but also its transporter activity [4,5].

## 2. Literature Review Procedure

First, we identified publications that discussed human placenta transporters that were published before May 2021 using the search term “transporters in human placenta” in PubMed. From those, we selected transporters that are able to recognize drugs as their substrates and that included data from humans and animals. These selected “drug transporters existing in the human placenta” are listed in Table 1. In the last step, we collected detailed information on validation in human cells or tissues. In the table, we have noted when a transporter has no substrate information available from human cells or tissues.

In the text and the table, we use the transporter acronyms that were used in the underlying original papers. Additionally, we provide the official gene name as per human gene nomenclature (HGNC).

## 3. Physiological Functions of Drug Transporters

Drug transporters have other functions and transport drugs because of their similarities with physiological substrates. Therefore, we provide a brief overview of the physiological functions and main expression sites (apart from the placenta) of the drug transporters that are described in this review.

### 3.1. ABC Transporters

The ABC transporter superfamily is a group of transporters that discharge drugs from the inside of cells to the outside of cells using ATP hydrolysis energy. Although they were originally identified as a multidrug resistance factor from tumor cells, they are also expressed in normal tissues and are able to transport a variety of drugs with different chemical structures and mechanisms of action (e.g., anticancer drugs, immunosuppressants, and inotropic glycosides). ABC transporters can be classified into seven subgroups, ranging from ABCA to G. Among these, ABCB, C, and G are recognized as drug transporters. The ABC transporter family has been studied more in depth than the SLC family [6]. The functional proteins of eukaryotic ABC transporters typically contain two nucleotide-binding domains (NBDs) and two transmembrane domains (TMDs). Eukaryotic ABC transporters are classified into two types: full transporters (ABCB1, ABCB4, and ABCC) and half transporters, which form either homodimers or heterodimers to constitute a functional transporter (ABCG). The genes of ABC transporters are highly conserved between species, indicating that most of these genes have existed since the beginning of eukaryotic evolution.

#### 3.1.1. ABCB (Multidrug Resistance: MDR) Family

ABCB1 (MDR1), which was initially called P-glycoprotein (P-gp), is expressed in the canalicular membrane of hepatocytes, renal proximal tubules, the luminal side membranes of the capillary bile ducts in the liver, the apical surface of intestinal enterocytes, especially in the distal ileum, and in the capillary endothelial cells in the brain and testis. MDR1 recognizes a wide range of lipophilic substrates. In the gastrointestinal mucosa, MDR1 drains substrates to the luminal side, and in cerebrovascular endothelial cells, MDR1 regulates the substrate distribution into the brain tissue [6,7,8,9]. ABCB4 (MDR3) acts as a flippase for phosphatidylcholine, a phospholipid that forms cell membranes, and changes the composition of the phospholipids that are present in the cell membranes. In the liver, MDR3 secretes the phosphatidylcholine from the hepatocytes into bile, and an MDR3 deficiency leads progressive familial intrahepatic cholestasis (PFIC) type 3 [10,11]. 

#### 3.1.2. ABCC (Multidrug-Resistance-Associated Protein: MRP) Family

ABCC1 (MRP1) is widely expressed throughout the body and is suggested to be involved in antioxidant activity [12,13]. ABCC2 (MRP2) is predominantly present in the liver and gallbladder, and its loss of function causes Dubin–Johnson syndrome [14]. ABCC3 (MRP3) is widely present throughout the body. In the placenta, MRP3 may be responsible for the transport of organic cholephilic anions [15]. ABCC4 (MRP4) is mainly expressed in the prostate and in the proximal tubules in the kidney and excretes prostaglandin [16]; ABCC5 (MRP5) is expressed throughout the body and excretes cyclic nucleotides to the outside of cells [17]; and ABCC6 (MRP6) is present in the liver and kidneys. MRP6 deficiency is the cause of “pseudoxanthoma elasticum”, which promotes ectopic calcification [18]. ABCC11 (MRP8) is predominantly expressed in the liver, testis, and prostate.

#### 3.1.3. ABCG2 (Breast Cancer Resistant Protein: BCRP)

ABCG2 (BCRP) transports uric acid and a wide variety of organic anions into the gastrointestinal tract, bile, and urine [19].

### 3.2. SLC Transporters

This group of transporters is responsible for secondary active transport and some passive transport. A large number of SLC transporters have been identified, with more than 400 transporters in over 60 families. Unlike ABC transporters, SLC transporters are highly diverse in structure and consist of a variety of folds. The information on the phylogenetic analysis of SLC transporters is also scarce due to a large number of subfamilies and their complex genetic structures [20,21].

#### 3.2.1. SLCO (Organic Anion Transporting Polypeptide: OATP) Family

SLCO1A2 (OATP1A2) is mainly expressed in the brain and transports thyroid hormone. SLCO 1B1 (OATP1B1) is responsible for the uptake of bile acids into the hepatocytes. SLCO2B1 (OATP2B1) is widely expressed in organs throughout the body and carries estrone 3 sulfate and dehydroepiandrosterone sulfate. SLCO3A1 (OATP3A1) is upregulated in the liver during cholestasis and contributes to the elimination of the bile acids that accumulate in the liver [22]. SLCO4A1 (OATP4A1) is widely present throughout the body and transports bile acids and thyroid hormones [23].

#### 3.2.2. SLC22A Family

This family includes three subfamilies: organic cation transporter (OCT), organic cation transporter novel type (OCTN), and organic anion transporter (OAT).

SLC22A1 (OCT1) is mainly expressed in the liver and is responsible for the uptake of dopamine and other substances into the liver. In the placenta, OCT1 transports acetylcholine as a substrate [24]. SLC22A2 (OCT2) is responsible for creatinine transport (efflux) in the kidney [25]. SLC22A3 (OCT3), also called extraneuronal monoamine transporter (EMT), transports monoamine neurotransmitters such as serotonin [26], norepinephrine, and dopamine and is present in more diverse tissues than OCT1 and OCT2.

SLC22A4(OCTN1) and SLC22A5 (OCTN2) are physiologically crucial transporters for L-carnitine. It has been proposed that OCTN2 plays a major role in L-carnitine transport from the mother to the fetus across the placenta [27]. SLC22A6 (OAT1) is highly expressed in the kidney and is responsible for the first step in the uptake of PGs and other substances from the blood into cells and their elimination into urine [28]. SLC22A11 (OAT4) is mainly found in kidney and placenta [28]. It may act to prevent potentially harmful organic anions from reaching the fetus. SLC22A13 (OAT10) is highly expressed in kidney and is responsible for uric acid transport. Decreased OAT10 function increases the risk of gout [29].

#### 3.2.3. SLC29A (Equilibrative Nucleotide Transporter: ENT) Family

Both SLC29A1 (ENT1) and SLC29A2 (ENT2) are expressed in a wide range of organs, while SLC29A4 (ENT4) is highly expressed in the adipose tissue. ENT1 and ENT2 are involved in the uptake of nucleic acids into the cells [30].

#### 3.2.4. SLC47A (Multidrug and Toxin Extrusion: MATE) Family

SLC47A1(MATE1) is present in a wide variety of organs, including the liver and kidney, while SLC47A2 (MATE2) is mainly expressed in the kidney. They excrete substrates into the urine and bile through the exchange transport of H+ and organic cations [31].

## 4. Morphology of the Human Placental Barrier

**Figure 1:**
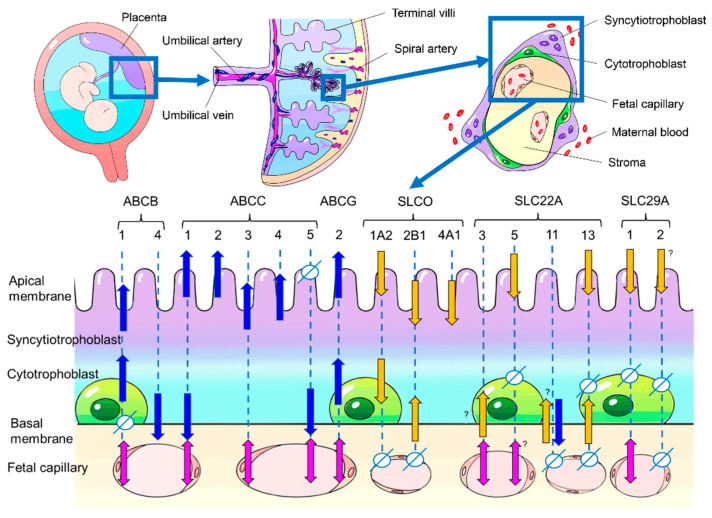
Human placental structure (above) and localization of drug transporters in human placenta (below). The blue arrows indicate the efflux direction, while the yellow arrows indicate the uptake direction in regard to the specifically shown cell type or compartment. The actual drug transport direction (discharge from placenta into the maternal circulation or uptake into placenta and fetal circulation) depends on the localization of the transporters. The transport direction is indicated by blue arrows for discharge and yellow arrows for uptake. Transporters in the fetal capillary are indicated by pink double-headed arrows because they may work in both directions depending on their exact localization in endothelium. The symbol “Φ” indicates a localization where the respective transporter does not exist. No symbols and no arrows mean that information is not available. The question mark symbol “?” shows that conflicting information exists.

The human placenta has a hemochorial structure, where fetal-derived villous trees are surrounded by maternal blood. The cellular layers of villi consist of, from the maternal blood side, syncytiotrophoblast, cytotrophoblast cells, stroma cells, and fetal vascular endothelium, which act to separate the fetal circulation from the maternal circulation (Figure 1). Several mechanisms are involved in the transport of substances across the placenta, from simple diffusion to selective transport by membrane transporters.

## 5. Drug Transporters Reported in Human Placenta

In the human placenta, 28 proteins have been reported to function as drug transporters. For each transporter, we show its localization in the placenta and its expression in each trimester of pregnancy and in the cell lines that are frequently used in transporter studies (Table 1) (Figure 1).

**Table 1:** Detailed information on drug transporters in the human placenta. For each transporter, the following information is given: name (both original and HGNC), physiological substrates, drug substrates, localization in human placenta, change in expression level with gestational week, and expression in representative cell lines. Conflicting reports on the localization of transporters are indicated with an asterisk (*). Although the transporters are expressed in the human placenta, some functional data are only available from animals (indicated by **) or from human cell lines or organs other than the placenta (listed in parentheses after drug names). For OATP4A1, there is no substrate whose uptake has been clearly evaluated. However, it is generally believed to be involved in drug transport, as is the case with other OATPs. Abbreviations: NA: no data available, ND: not detected, R: RNA was detected, P: protein was detected, Sy: syncytiotrophoblast, Cy: cytotrophoblast, FE: fetal endothelium, LTC4: leukotriene C4, E217bG: 22C10–3 estradiol 17b D glucuronide, PG: prostaglandin, “+”: respective cells or tissues are positive.

Superfamily	Family	Transporter Name	HGNC Name	Physiological Substrates	Drug Substrates	Localization in Placenta			Period (Trimester)				Cell Lines				References
						Sy	Cy	FE	1st	2nd	3rd	Unknown	BeWo	JEG3	JAR	HTR	
ABC	ABCB	MDR1	ABCB1	hydrophobic compounds	vinblastine, vincristine, digoxin, saquinavir	+	+	+	R, P	R, P	R, P		+	+	+	+	[15,32,33,34,35,36,37,38,39,40]
		MDR3	ABCB4	bile acids	vinblastine, digoxin	+	NA	NA	R	NA	R		+	+	+	NA	[11,15,33,37]
	ABCC	MRP1	ABCC1	bile acids, folic acid, LTC4, E217bG,	maraviroc, pravastatin	+	ND	+	R	NA	R, P		+	+	+	+	[37,41,42,43,44,45,46]
		MRP2	ABCC2	organic anion	talinolol	+	NA	NA	R	R, P	R, P		+	+(P)	+	NA	[15,42,44,45,47,48,49,50,51]
		MRP3	ABCC3	cholate	methotrexate (HEK293)	+	ND	+	R	NA	R, P		+	+	+	NA	[15,42,44,45,48,52]
		MRP4	ABCC4	estradiol, cAMP, cGMP,	adefovir (kidney)	+	NA	NA	NA	NA	R, P		+	+	+	NA	[15,48,53]
		MRP5	ABCC5	cAMP, cGMP	doxorubicin (nonsmallcell lungcancer cell-lines)	+	ND	+	R	NA	R, P		+	NA	NA	NA	[44,45,54]
		MRP6	ABCC6	LTC4	etoposide, doxorubicin, BQ−123 **	NA	NA	NA	NA	NA	NA	R	NA	NA	NA	NA	[18,55,56]
		MRP8	ABCC11	cGMP, cAMP	maraviroc	NA	NA	NA	NA	NA	R		+	+	+	NA	[15,57,58,59,60]
	ABCG	BCRP	ABCG2	organic anion	pravastatin, nitrofurantoin	+	+	+	R, P	R, P	R, P		+(P)	+(P)	+	NA	[15,35,37,38,40,50,51,61,62,63,64]
SLC	SLCO	OATP1A2	SLCO1A2	unconjugated bilirubin, steroids, thyroid hormones	maraviroc	+	+	ND	R, P	NA	R, P		+	+	+	NA	[11,15,41,65]
		OATP1B1	SLCO1B1	estradiol, taurocholate, leukotrienes, steroids, thyroid hormones	rifampicin (kidney), pravastatin (HEK293)	NA	NA	NA	R	NA	ND		ND	ND	ND	NA	[11,15,66,67]
		OATP2B1	SLCO2B1	estrone 3-sulfate	fexofenadine	+	+	NA	NA	NA	R		+	+	+	NA	[11,15,68,69]
		OATP3A1	SLCO3A1	vasopressin, PG, thyroid hormones	simvastatin (HEK293)	NA	NA	NA	R	NA	R		NA	NA	NA	NA	[11,68,70]
		OATP4A1	SLCO4A1	taurocholate, PG	**	+	ND	ND	R, P	NA	R, P		NA	NA	NA	NA	[11,65,71]
	SLC22A	OCT1	SLC22A1	choline, dopamine	metformin (HEK293), pazopanib(hepatocytes), ranitidine (HEK293)	NA	NA	NA	NA	NA	R		+	+	NA	NA	[24,27,28,72,73,74,75,76]
		OCT2	SLC22A2	histamine, dopamine,	metformin (HEK293)	NA	NA	NA	NA	NA	R		ND	±	NA	NA	[24,27,72,76,77]
		OCT3	SLC22A3	organic cations	metformin (HEK293)	+ *	+ *	+	R, P	R, P	R, P		ND	ND	NA	NA	[26,27,72,76,77,78,79]
		OCTN1	SLC22A4	carnitine, organic cations	sulpiride **	NA	NA	NA	NA	NA	R		+	+	NA	NA	[27,80]
		OCTN2	SLC22A5	carnitine, organic cations	etoposide (HEK293), quinidine, verapamil, and valproate (HEK293)	+	ND	+ *	NA	NA	R, P		+	+	NA	NA	[27,81,82,83,84]
		OAT1	SLC22A6	alpha -ketoglutarate, PGE2, PGF2a, cGMP, cAMP	adefovir (kidney)	NA	NA	NA	NA	NA	NA	R	NA	NA	NA	NA	[85]
		OAT4	SLC22A11	estrone 3-sulfate,	olmesartan	+	+	ND	R, P	R, P	R, P		+	+	NA	NA	[51,68,86,87,88,89,90]
		OAT10	SLC22A13	urate, organic cations	cyclosporine **	+	ND	ND	NA	R, P	R, P		+	NA	NA	NA	[88,91,92]
	SLC29A	ENT1	SLC29A1	adenosine, inosine	entecavir, abacavir	+	ND	+	R	NA	R, P		+	NA	NA	NA	[93,94,95,96,97]
		ENT2	SLC29A2	adenosine, inosine	entecavir	+	ND	ND	R	NA	R, P		+	NA	NA	NA	[93,94,96,97]
		ENT4	SLC29A4	dopamine, histamine, adenosine	atenolol (HEK293)	NA	NA	NA	NA	NA	R		NA	NA	NA	NA	[73,98]
	SLC47A	MATE1	SLC47A1	creatine, thiamine	metformin (HEK293), cimetidine (HEK293)	NA	NA	NA	R	NA	R		NA	NA	NA	NA	[76,99,100]
		MATE2	SLC47A2	creatine, thiamine	metformin (HEK293), aciclovir (HEK293)	NA	NA	NA	R	NA	R		NA	NA	NA	NA	[76,99,101]

### 5.1. ABC Transporters

#### 5.1.1. ABCB (MDR) Family

ABCB1 (MDR1) is the transporter that was identified first and is the one that has been the most intensively studied. The expression of MDR1 decreases with progressing gestational age, while the expression of ABCB4 (MDR3) increases [11,34]. The expression level of MDR1 in BeWo cells, which are a frequently used model of human syncytiotrophoblast, is lower than it is in the human placenta, including in term placenta [37,40,73].

#### 5.1.2. ABCC (MRP) Family

ABCC3 (MRP3) is highly expressed in the placenta, but it is express much less in cell lines, whereas ABCC11 (MRP8) is expressed more in cell lines [15]. It was cloned relatively recently, and data are scarce.

#### 5.1.3. ABCG2 (BCRP)

This transporter has been comparatively well studied and is widely expressed in both the placenta and in cell lines. ABCG2(BCRP) is expressed in the apical side of syncytiotrophoblasts, cytotrophoblasts, and fetal endothelial cells. In BeWo, the expression level is higher than it is in the placenta [63]. Although there are various reports on a correlation with the expression level with gestational age, BCRP does not seem to change significantly or decrease slightly as pregnancy progress [36,38,50].

### 5.2. SLC Transporters

#### 5.2.1. SLC21A (OATP) Family

This group of transporters has been isolated from various animal species, including humans. However, they have more diversity among species than other drug transporters and have many isoforms without homologues between species. SLC21A9 (OATP2B1) expression is far less abundant in cancer-derived cell lines, including in the most frequently used choriocarcinoma cell lines [15].

#### 5.2.2. SLC22A Family

Although SLC22A1 (OCT1) and SLC22A2 (OCT2) have been reported to be expressed in very small amounts, it is commonly recognized that SLC22A3 (OCT3) is the only OCT family member that is clearly expressed in the human placenta, but there are conflicting reports on its cellular localization. One study reported OCT3 expression in the basal membranes of syncytiotrophoblast [78,79], while Kliman et al. showed its exclusive expression in cytotrophoblast cells by IHC [26]. Liu et al. classified trophoblasts into subtypes using single-cell RNA sequencing and analyzed the imprinted gene expression. In this report, OCT3 appears to be expressed in a CTB-dominant manner [102]. Single cell transcriptome research in the human placenta is summarized in a review by Li et al. [103]. For SLC22A5 (OCTN2), different localizations have been reported in fetal blood vessels [81,82]. As described above, the SLC transporters basically act in the direction of substrate uptake, while SLC22A11 (OAT4) also excretes substrates. However, the involvement of this “efflux mode” in drug transport has not been sufficiently proven [91]. OAT4 is expressed in the JEG3 choriocarcinoma cell line but only when forskolin is added [89].

#### 5.2.3. SLC29A (ENT) Family

SLC29A1 (ENT1) is expressed on the apical side of the syncytiotrophoblasts, while SLC29A2 (ENT2) is expressed at lower concentrations [93,94,96,97].

#### 5.2.4. SLC47A (MATE) Family

The placental expression levels of both SLC47A1 (MATE1) and SLC47A2 (MATE2) decrease as the gestational weeks progress [99].

## 6. Regulation of Placental Drug Transporters

When considering the functional effects of drug transporters, the expression level of the transporters is also an important factor. As mentioned above, the expression levels of some transporters have been shown to change with the number of gestational weeks. In addition, some factors such as drug–drug interactions, some kinds of pathological conditions such as GDM or preeclampsia, and genetic variations such as single-nucleotide polymorphisms (SNPs) have been reported to cause changes in the expression levels of these transporters. The transporter regulation is summarized in the review by Staud and Ceckova [104].

## 7. Experimental Models for Human Placental Drug Transport

### 7.1. Cell Lines

The most commonly used cell line in studies on human placental transport is the choriocarcinoma cell line BeWo, which forms a confluent monolayer with the microvilli on a permeable filter support. BeWo cells are often used as a model for trophoblast maturation because morphological and biochemical changes and syncytialization occur when the intracellular cAMP concentration is increased due to stimulation with forskolin. However, it should be noted that the syncytialization of BeWo may cause changes in the permeability of the monolayer due to the widening of the intercellular spaces and increases in the transport of certain substances [105]. After BeWo, the most commonly used cell line is the choriocarcinoma-derived cell line JEG3. The addition of forskolin only alters JEG3 biochemically, but it does not form a syncytium. The choriocarcinoma cell line JAR is more similar to pre-differentiated cytotrophoblast cells and is less commonly used for transporter studies. Further, HTR-8/SVneo is a cell line that was established by immortalizing extravillous trophoblasts, which are derived from cytotrophoblasts. Their characteristics do not sufficiently reflect those of physiological trophoblast cells, and therefore, they are rarely used in transporter studies [106]. Cell lines are frequently used for functional analyses because they are easy to handle and can be used for overexpression and inhibition studies, as described below.

### 7.2. Placental Explants

Placental explants are less frequently used for transport studies than cell lines because syncytiotrophoblasts degrade rapidly, but they can be used for functional and toxicological analyses [71]. Placental explants have the advantage of reflecting the physiological villous structure. The localization of cells and factors of interest can be easily determined by immunohistochemistry and other methods [107,108]. 

### 7.3. Placental Membrane

The so-called isolated “membrane vesicles” from term placenta have often been used in transporter studies. Previously, they were isolated by means of the apical membrane preparation [109] and basal membrane preparation [110] methods and are mainly used to determine the localization of various transporters. However, as seen in OCT3, there are some cases where membrane preparation and IHC have demonstrated different results.

### 7.4. Placenta-on-a-Chip

Recently, microfluidic systems called “organs-on-a-chip” have been actively studied, including placenta-on-a-chip. To simulate the placental barrier, co-cultures of BeWo or HTR−8/SVneo and Human Umbilical Vein Endothelial Cells (HUVEC) or Human Placental Vascular Endothelial Cells (HPVEC) have been commonly used [111,112]. So far, the use of primary trophoblast cells in this model has not been published. Placenta-on-a-chip technology is still in its beginning, and only a few studies have been reported. However, it has potential for use in the evaluation of drug transport, as previously reported [113], and further development is expected.

### 7.5. Ex Vivo Placenta Perfusion Model

This model has been commonly used in toxicological studies. Unlike the in vitro models, which are far from the real in vivo situation, the main feature of this model is its ability to reproduce macroscopic transport in human placenta. The limitations of this model are its short maintenance time of 6 h, and situations modelling the first trimester cannot be reproduced. Nonetheless, this model is a human organ models that can mimic the in vivo situation very closely. Indeed, May et al. perfused candidate transporter inhibitors with talinolol and evaluated the transporters that were involved in the materno–fetal transfer of talinolol [47].

### 7.6. Functional Assays on Placental Drug Transporters

During functional transporter analyses, cell lines are frequently used. A common method is to add the inhibitors of specific transporters and to evaluate the changes that take place in terms of the substrate uptake [37,41,47,61]. This method has also been performed with placental explants [86]. The role of specific transporters during substrate uptake in cell lines can also be evaluated by overexpression or knock down using RNA mimics or siRNA [46]. Cell lines may also be used to establish hypotheses that need further confirmation in primary cells or tissues. However, there are significant differences in the expression of drug transporters between cell lines and physiological placental tissues or cells [95].

## 8. Discussion

In this review we have summarized the findings related drug transporters in the human placenta and in experimental models. Transporters have been intensively studied in the kidney, liver, gastrointestinal tract, and at the blood–brain barrier. Pharmacokinetic knowledge is vital in terms of drug efficacy and side effects. 

Additionally, the placenta has a strong barrier function in order to ensure that maternal and fetal blood avoid contact with each other and do not mingle, but simultaneously, it has a very active transport function that is used to aliment the fetus and to eliminate its waste products. However, from the studies that have been published thus far, most do not explain the complete mechanisms of transportation of particular substances from the maternal surface to fetal circulation. Understanding the placental permeability of drugs is very important for estimating their toxicity to the fetus as well as for the pharmacological treatment of the fetus.

There are several difficulties in studying drug transporters. First, in vitro models have limitations, as shown by the different transporter localization reuslts in the human placenta when the membrane preparation method or immunostaining have been used (e.g., for OCT3 [26,78,79]). Since l transporter localization is a very important aspect for elucidating the whole picture of substrate uptake, this issue should be considered as an area of focus in the future. Second, there are many further factors to be considered in vivo, such as binding proteins and competing substrates. As it is difficult to reproduce several factors in vitro or in ex vivo models simultaneously, their results do not completely reflect the in vivo situation. The third point is the broad substrate specificity of different transporters. It is not rare that a single substrate is recognized by multiple transporters in the same organ. In other words, the recognition of a drug by a certain transporter is not equal to its importance in pharmacokinetics.

Despite the technical challenges in studying placental transporters, the continuous improvement of experimental models and the combination of different types of experimental methods will lead to better understanding. Developments in the knowledge of placental drug transporters will be beneficial for the health and optimization of drug treatment for pregnant women and their fetuses.

## 9. Conclusions

In this review, we described the functions and the expression and localization of drug transporters in the human placenta and in experimental models that have been previously used for their study. Detailed knowledge is fundamental for planning drug applications in pregnancy as well as for the consideration of the potential side effects of those applications.
